# Development of New Hybrid Composites for High-Temperature Applications

**DOI:** 10.3390/polym15224380

**Published:** 2023-11-10

**Authors:** Rubén Seoane-Rivero, Lorena Germán, Fernando Santos, Koldo Gondra

**Affiliations:** 1 GAIKER Technology Centre, Basque Research and Technology Alliance (BRTA), Parque Tecnológico de Bizkaia, Edificio 202, 48170 Zamudio, Spain; 2 Fundación AZTERLAN, Basque Research and Technology Alliance (BRTA), Aliendalde Etxetaldea 6, 48200 Durango, Spain

**Keywords:** FML, composite, epoxy, carbon fiber, aluminum, temperature

## Abstract

Nowadays, in the automation and aircraft industries, there is a challenge in minimizing the weight of components of vehicles without losing the original properties. In this study, we fabricate hybrid composites based on fiber metal laminates; these materials could be promising composites for high-performance applications. This work is focused on analyzing the effect of high temperature (175 °C) on the mechanical properties of these kind of materials, by introducing NaOH and silane adhesion treatments between metal and prepreg layers and by using vacuum molding processes. Fabricated FML (NaOH treatment) shows a significant improvement in tensile strength in comparison with the ARALL and GLARE reported by ESA. Moreover, developed FMLs at 175 °C kept more than 70% of their tensile strength and modulus and kept 4% of tensile strain at room temperature. The prominent conclusion achieved in this work has been that excellent candidates have been obtained for a wide range of applications, including but not limited to space and aerospace applications.

## 1. Introduction

The demand for lightweight, strong and durable structures has increased in recent years [1,2]. That is why fiber metal laminates are gaining popularity in different applications: automotive, aircraft and space [3,4,5,6]. Fiber metal laminates (FMLs) are hybrid composite structures based on thin metal sheets and layers of fiber-reinforced resin. The main advantages of FMLs are an excellent fatigue resistance, damage tolerance, and impact resistance compared to monolithic metal alloys [7,8].

The reinforcement in the composite material is the fiber. In the case of FMLs, there are several types of fibers that can be used as reinforcement. In the 1980s, aramid-fiber-reinforced aluminum alloy laminates (ARALLs) [9,10] were implemented in the aerospace industries due to their impact strength, fatigue resistance and anti-corrosive nature that remained stable. In the late 1990s, glass-reinforced aluminum laminates (GLAREs) [11,12,13] were used in the aerospace industry due to their excellent properties. According to the cited literature study, glass fiber produces good results in mechanical properties [14,15]. Glass fibers are used as a reinforcing agent for many polymeric products to form a very strong fiber-reinforced polymer (FRP) composite material [16]. Multiple parts of American C-17 aircraft, Airbus A380 and aircraft fuselages were fabricated using ARALL and GLARE FMLs as a new structural material [17,18,19,20]. It is important to note that carbon-fiber-reinforced aluminum laminates (CARALLs) have received less attention than the previous ones. Despite their excellent impact resistance [21,22], one of the main disadvantages is their harder production process [23]. CARALLs provide superior material properties compared to metals and thus enable lighter structural designs to be achieved [24,25,26,27]. For space applications, these types of FMLs are usually fabricated by continuous carbon fiber with an epoxy matrix. The possibility of providing a structural performance with a very low weight, which represents important economic savings, has been the main driver for the use of reinforced polymer composites with carbon fiber [28,29,30,31].

Nowadays, the scope of these investigations is to find the behavior of aluminum foils to increase the mechanical properties [32,33], such as tensile strength and impact resistance; for example, the study conducted by Dhanaraj investigates the applicability of glass fibers as a reinforcement material for structural elements through experimental characterization, such as durability and mechanical properties (bending) [34]. To increase good bonding and adhesion between the fiber, metal and epoxy resin, circular holes with a 3 mm diameter were drilled in an aluminum foil at a pitch distance of 25 mm.

Epoxy resin [35,36] has an excellent adhesion to different materials, high strength, toughness, resistance to chemical attack, humidity, moisture resistance, better electrical insulation property, is odorless, is non-toxic, has a negligible shrinkage, etc. This resin reacts with itself in the presence of catalysts or with many co-reactants, such as amines, phenol, thiol, thiols, etc. Epoxy resin has many industrial applications for a variety of purposes. It has higher mechanical properties and more thermal and chemical resistance than other types of resin. Therefore, it has an important use in the manufacture of aircraft [37,38] and space [39,40] components.

To carry out the preparation of these FML composites, carbon fiber epoxy prepreg and aluminum foils have been used It is important to note that fabricated FMLs demonstrated promising properties due to adhesion treatments, and the FMLs at 175 °C maintained more than 70% of their tensile properties at room temperature.

## 2. Materials and Methods

### 2.1. Materials

The prepreg used for the study was MTM^®^46-38%-12KT700SC60E-2X2T-660-1250, (Cytec Engineered Materials (Wrexham) Ltd., Sinclair, Heanor, UK) which meets the ESA (European Space Agency) outgassing requirements under the ECSS-Q-ST-70-02C regulation. This material was manufactured by Cytec Engineered Materials. This prepreg is composed of high modulus carbon reinforcement and epoxy resin. This prepreg exhibits an excellent retention of Tg under wet conditions, and it can be processed via low pressure vacuum-bag Out-of-Autoclave (OoA) molding or autoclave molding. Moreover, it can be cured at temperatures as low as 80 °C, allowing the use of low cost tooling for prototypes and short production runs. This material was purchased from Solvay. The aluminum used was AW6082, which was supplied by Alu-stock. This aluminum is a medium-strength alloy with an excellent corrosion resistance and high mechanical properties. Sodium hydroxide (≥97%) was purchased from Sigma Aldrich, and Chemlok 144 from Lord. All materials were used as received.

### 2.2. Ansys Simulation

It is important to note that prior to the development of the manufacturing process, a simulation of the stresses generated in the hybrid material due to the different thermal expansion coefficients of the materials when subjected to thermal cycles in a simplified 2/1 structure, formed by two layers of aluminum and one of carbon composite, was carried out. The stresses generated in both the carbon composite and the aluminum have been analyzed when increasing the temperature up to 175 °C. The thickness corresponding to the aluminum sheets was 1 mm, and that of the prepreg sheet was 0.6 mm. The parameters used in the simulation of the FML composite are shown in Table 1.

### 2.3. Measurements

A scanning electron microscopy (SEM) Zeis EVO 50 microscope at 20 kV and an energy dispersive X-ray (EDX) (INCA, Oxford Instruments, Abingdon, Oxfordshire) were used to analyze morphological and elemental compositional data, respectively. All samples were gold-palladium-coated by a sputter Leica EM SCD005 before the measurements. Moreover, a 3D optical profiler PLμ NEOX (Sensofar, Barcelona, Spain) was used to analyze the surface topography.

A universal testing machine (Autopraph AG-X 100kN, Shimadzu, Kyoto, Japonia) was used to obtain the mechanical properties of the FML composites, including the modulus and flexural strength. Samples for flexural measurements were prepared according to UNE EN ISO 14125 and loaded to fail at a speed rate of 2 mm/min for flexural measurements. The sample dimensions were 12 × 1.5 × 3 mm. In the case of tensile tests, samples were loaded to fail at a speed rate of 2 mm/min. The rectangular samples’ dimensions were 10 × 1 × 3 mm according to ASTM B557-15. It is important to note that a thermal chamber was used for mechanical testing at 175 °C; samples were introduced in the chamber at 175 °C 30 min before testing. In both cases, measurements were carried out with a load cell of 5 kN at room temperature and 175 °C. For each composite, three samples were tested, and the average value was reported.

The sanding cycles performed on the surface of the aluminum were: 20, 60 and 120 cycles. In order to observe its effect on the surface, confocal microscopic characterization was carried out. One of the problems encountered in the manufacture of the FML materials was a poor adhesion, mainly due to the different thermal expansion coefficients of the materials: aluminum and epoxy prepreg with carbon fiber. Therefore, we proceeded to the study of different treatments of the sanded aluminum by chemical attack with NAOH and silanes. The morphology and composition generated on the surface of the aluminum was analyzed by confocal microscopy studying the topography (roughness) and SEM-EDX microscopy analyzing the surface composition.

### 2.4. Fabrication of FML Composites

To carry out the processing of the FMLs, the following symmetrical arrangement was carried out: aluminum 6082 foil—3 layers of prepreg (reference MTM 46)—aluminum 6082 foil. The aluminum sheets are preconditioned with the aid of an automatic sander (P400 sandpaper) in order to facilitate adhesion between layers. A vacuum molding press (-SUB 3D, Global vacuum presses) was used for manufacturing plane coupons of hybrid material. The material temperature was monitored by themopar. Composite plates were fabricated and were 300 mm long and 300 mm wide, with a thickness of around of 3 mm.

**Figure 1:**
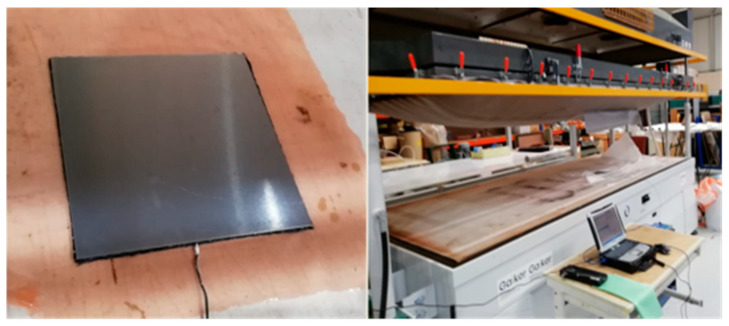
The vacuum press curing process.

First, the specimens are placed on the press bed, and pressure is exerted on them with the aid of a vacuum membrane as can be seen in Figure 1. Once the desired vacuum is generated, the (progressive) heating process is started until the target temperature is reached. Then, the material was cooled before removing the coupon from the machine.

## 3. Results

**Figure 2:**
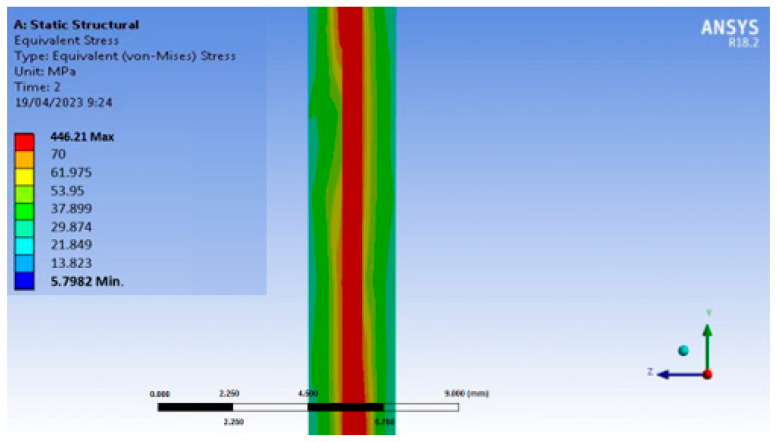
Ansys simulation of FML composite.

The stresses generated in both the carbon composite and the aluminum are shown in Figure 2.

The following table shows the maximum tensile stresses generated in different materials present in the FML when this hybrid material is heated between room temperature to 175 °C. These data were obtained by simulation and compared with the tensile strength of the aluminum and the epoxy composite, individual materials, at 175 °C, which were obtained in tests carried out in the laboratory (see Table 2).

In all cases, the maximum stresses suffered in the heating studied with Ansys do not exceed the maximum strength of the individual materials under these conditions, so it can be expected that if the adhesion between aluminum and metal is good, the FML will withstand the temperature changes.

In order to ensure the correct adhesion between aluminum and epoxy composite, as a first approximation, different attacks through wear processes with sandpaper measuring 600 and 1000 were carried out.

**Figure 3:**
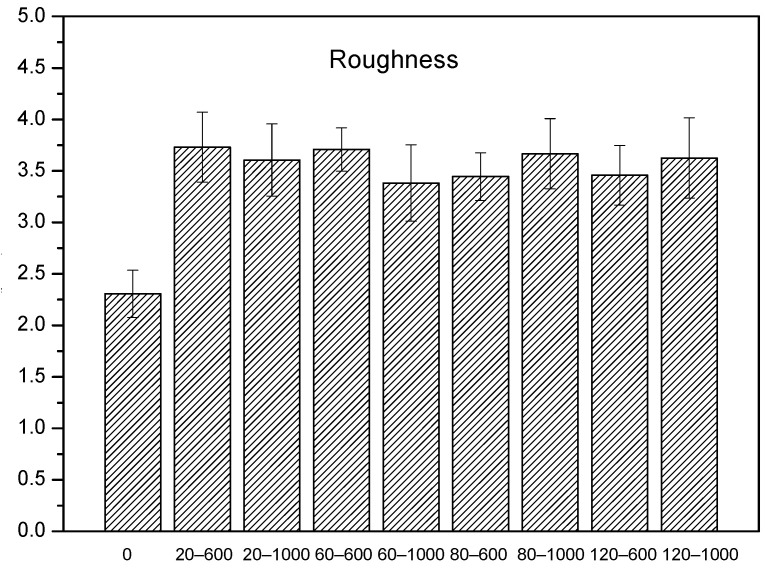
Roughness of Al after different sanding cycles.

After carrying out the characterization, it was observed that the surface roughness did not decrease with the increase of the wear cycle; thus, taking into account this property, we defined 20 wear cycles as the optimum, since the roughness hardly varied with a higher number of cycles, as can be seen in Figure 3.

**Figure 4:**
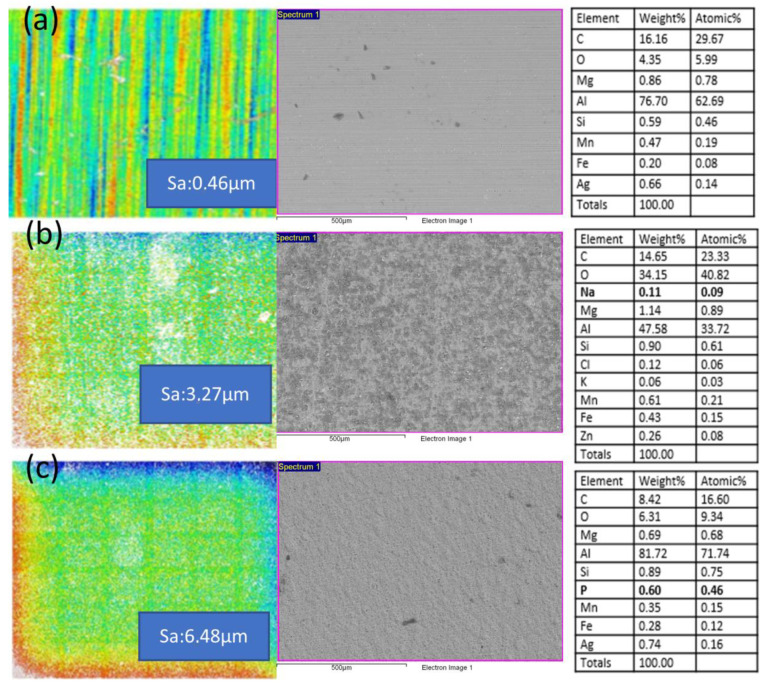
Confocal microscopy and SEM-EDX images of the analyzed Al surfaces. (**a**) Reference sample, (**b**) NaOH chemical attack, and (**c**) silanes chemical attack.

The treatments carried out not only chemically modify the surface but also increase the roughness, which will facilitate mechanical adhesion between the two materials. As can be seen in Figure 4, the elements incorporated in each treatment are detected, verifying their deposition on the surface and therefore certifying the modification of the chemical composition of the aluminum interface.

**Figure 5:**
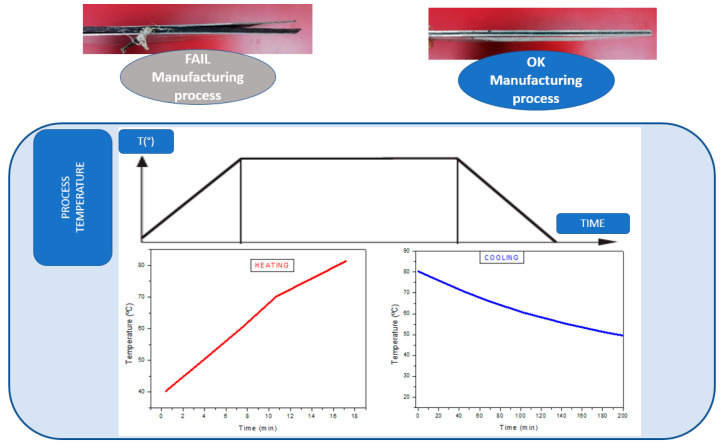
FML manufacturing process. Temperature monitoring graphics.

Once the stress simulation study on the hybrid material was carried out, the different processes were compared, and the vacuum press curing was optimized. The fine-tuning of the FML manufacturing process in the vacuum press focused on the following points, as can be seen in Figure 5:

- Adjustment of vacuum temperature and times in the three process stages: heating, curing and cooling.
- Monitoring of material heating. Temperature deviation from the heating program.

The process variables introduced into the vacuum press are as follows:

- Temperature: 80 °C (dome and bottom plate).
- Exerted pressure: 920 millibars.

First, the specimens are placed on the press bed, and pressure is exerted on them with the aid of a vacuum membrane. Once the desired vacuum is generated, the (progressive) heating process is started until the target temperature is reached at a heating rate of 3 °C/min. The samples are kept in the equipment for approximately 5 h. Finally, the cooling step is carried out, maintaining the vacuum pressure, at a cooling rate of 0.2 C°/min, and the specimens are allowed to cool for 2 to 3 h (the temperature drops to approximately 50 °C). FML composites were fabricated under the same vacuum (920 mmHg).

The mechanical characterization of the material developed in the project has been carried out by studying the flexural and tensile behavior of the materials and analyzing how extreme temperature affects the mechanical performance. This is why the mechanical tests were conducted at room temperature and 175 °C. It is observed that working at very high temperatures decreases the modulus and the tensile and flexural strength. However, the deformation that the material can withstand before failure is greater than at room temperature, as shown in the table below. The Table 3 and Table 4 show the average flexural modulus and strength of the fabricated FMLs. It was not possible to conduct tensile and flexural characterization without treatments; all samples broke down before starting the mechanical test.

**Figure 6:**
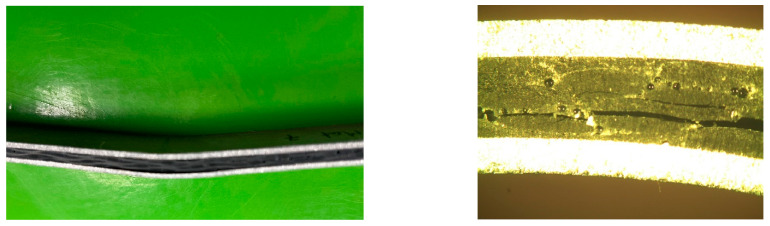
Flexural test piece.

It should be noted, as shown in Figure 6, that the prepreg and aluminum layers did not peel off, so there was not an adhesion failure between the composite and metal but a cohesive failure of the composite material. This characterization was carried out at 175 °C and room temperature.

In accordance with ESA documentation “Structural materials handbook ECSS-E-HB-32-20 Part 7A, the hybrid materials aluminum aramid fiber composite (ARALL) and aluminum glass fiber composite (GLARE) are used. The applications where these materials can be used are as follows. These hybrid materials are of particular interest for applications in shear panels and firewalls. If single curvature structures are required, the cold-forming capabilities of FML panels may be attractive, and they could also be attractive in applications where good acoustic damping is needed. In addition to these properties, the carbon FMLs developed in the project could provide an enhanced mechanical performance for different applications.

**Figure 7:**
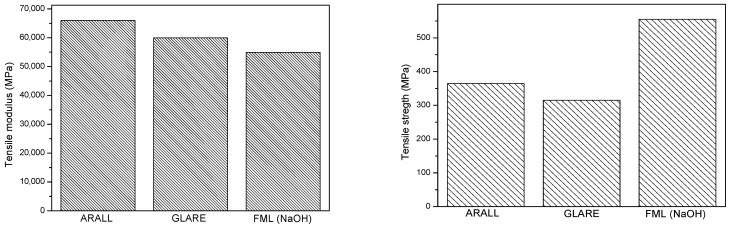
Comparison of tensile properties of ARALL, GLARE and fabricated FML.

Figure 7 compares the mechanical performance of the materials reported in ECSS-E-HB-32-20 Part 7A [41] with the properties of the material developed in this work. The characteristics of the compared materials are as follows:

- ARALL 2: 2024-T6/UD aramid/epoxy
- GLARE 3: 2024-T3/UD R-glass/epoxy
- FML (NaOH treatment): 6082/0/90 carbon/epoxy

The material developed in this work presents a significant improvement in tensile strength in comparison with the aramid and glass systems. However, the tensile modulus is slightly lower than the other two materials. As for the deformation capacity before breakage, the developed FML shows a significantly higher deformation than the aramid material and a lower one than the glass fiber system. The mechanical strength of the studied materials decreased when the test temperature increased until 175 °C, as was expected. However, the flexural deflection and tensile strain were not affected by this temperature rise, presenting around 3.5 mm of flexural deflection and around 4% of tensile strain.

These are preliminary results, and it will be necessary to continue with the study of aluminum-carbon hybrid material by analyzing its different structures and geometries, as well as its behavior against fatigue and vibration, amongst other factors.

## 4. Conclusions

In summary, we developed promising hybrid materials that could have a positive impact in diverse areas, including polymer and metal research areas. FML composites have been fabricated with different adhesion treatments by using the vacuum molding process. The structural changes related to adhesion treatments were clearly reflected by the SEM-EDX and confocal microscopy. In addition, the mechanical properties of the FML composites demonstrated promising properties in terms of the tensile and flexural modes at ambient and high temperatures. It is important to note that thanks to adhesion treatments, the FMLs at 175 °C maintained more than 70% of their tensile properties at room temperature. Based on these characteristics, the developed composites represent excellent candidates for a broad range of applications, including space and aircraft, among others.

## Figures and Tables

**Table 1:** Parameters used in Ansys simulation.

Property	Temperature	Aluminum	Epoxy Carbon Prepreg	
			Plane	Perpendicular to the Plane
**Density (kg·m^3^)**	RT	2770	1420	
**C** **oefficient of thermal expansion (°C^−1^)**	RT	23 × 10^−6^	2.2 × 10^−6^	10 × 10^−6^
**Young’s modulus** **(MPa)**	RT	71000	61300	6900
**Poisson’s ratio**	RT	0.33	0.04	0.3

**Table 2:** Results of the tensile stresses generated when heating the hybrid material obtained by simulation and tensile strength of individual material obtained by laboratory tests.

	Aluminum (Mpa)	Composite (Mpa)
**175 °C Tensile stresses (simulation)**	70	245
**175 °C Tensile strength**	241	578

**Table 3:** Tensile results.

	Tensile Strength/Mpa		Tensile Modulus/Gpa		Tensile Strain/%	
	Ambient temperature	175 °C temperature	Ambient temperature	175 °C temperature	Ambient temperature	175 °C temperature
FML NaOH treatment	530 ± 36	472 ± 29	52.7 ± 1.7	37.8 ± 9.4	4.0 ± 0.4	4.1 ± 0.5
FML Silane treatment	467 ± 77	333 ± 47	55.3 ± 4.2	52.7 ± 11.2	4.3 ± 0.1	4.4 ± 0.3

**Table 4:** Flexural results.

	Flexural Strength/MPa		Flexural Modulus/GPa		Flexural Deflection/mm	
	Ambient temperature	175 °C temperature	Ambient temperature	175 °C temperature	Ambient temperature	175 °C temperature
FML NaOH treatment	571 ± 13	132 ± 21	50.5 ± 1.6	15.1 ± 1.6	3.4 ± 0.3	3.5 ± 0.6
FML Silane treatment	586 ± 15	104 ± 15	48.9 ± 3.1	17.1 ± 7.6	3.5 ± 0.5	3.6 ± 0.5

## Data Availability

The data are available in the manuscript.
